# Assessment of the Characteristics of Waxy Rice Mutants Generated by CRISPR/Cas9

**DOI:** 10.3389/fpls.2022.881964

**Published:** 2022-06-10

**Authors:** Yuhao Fu, Tingting Luo, Yonghuan Hua, Xuehai Yan, Xu Liu, Ying Liu, Yiping Liu, Baoli Zhang, Rui Liu, Zizhong Zhu, Jun Zhu

**Affiliations:** ^1^Rice Research Institute of Sichuan Agricultural University, Chengdu, China; ^2^State Key Laboratory of Crop Gene Exploration and Utilization in Southwest China, Sichuan Agricultural University, Chengdu, China; ^3^Key Laboratory of Southwestern Chinese Medicine Resources, Chengdu University of Traditional Chinese Medicine, Chengdu, China; ^4^Leshan Municipal Bureau of Agriculture and Rural Affairs, Leshan, China

**Keywords:** apparent amylose content, waxy rice, amylopectin structure, Waxy gene, CRISPR/Cas9

## Abstract

The cooking and eating quality of rice grains is a major focus from a consumer’s perspective and is mainly determined by the apparent amylose content (AAC) of the starch. Waxy rice, a type of rice with an AAC of less than 2%, is an important goal for the breeding of high-quality rice. In recent years, the cloning of the *Waxy* (*Wx)* gene has revealed the molecular mechanism of the formation of waxy traits in rice. However, there have been limited studies on the physicochemical properties, such as gelatinization temperature, rapid viscosity analyzer profile, and amylopectin fine structure of *wx* mutants. In the current study, a rapid and highly efficient strategy was developed through the CRISPR/Cas9 gene-editing system for generating *wx* mutants in the background of five different rice varieties. The *wx* mutation significantly reduced the AAC and starch viscosity but did not affect the major agronomic traits (such as plant height, panicle number per plant, grain number per panicle, and seed-setting frequency). Incorporation of the *wx* mutation into varieties with low initial AAC levels resulted in further reduction in AAC, but without significantly affecting the original, desirable gelatinization traits and amylopectin structure types, suggesting that parents with low initial AAC should be preferred in breeding programs.

## Introduction

Starch consists of two classes of α-polyglucans, amylose and amylopectin. Amylose is primarily a linear polymer, consisting of 1,4-D-glucopyranosyl units, while amylopectin is a highly branched polymer (Hizukuri et al., 1981; Hizukuri, 1986), with the amylose: amylopectin ratio in conventional rice being approximately 20: 80. The amylose content (AC) of the starch is arguably the most important quality indicator in rice grains, particularly with respect to cooking, processing, and eating qualities (Juliano, 1998). Rice cultivars can be divided into five categories according to AC: waxy (0–2%), very low (5–12%), low (13–20%), intermediate (21–25%), and high amylose (26–33%) (Juliano, 1992). Waxy rice (or glutinous rice) is regarded as high-quality rice, which is usually sticky when cooked (Juliano, 1998). Because of its unique, functional characteristics, waxy rice is widely used in processed food, medicines, and cosmetics (Bao et al., 2004; Puchongkavarin et al., 2005; Chun et al., 2010).

Amylose is proposed to have a significant influence on the physicochemical properties of waxy rice flour (Jane et al., 1999). Waxy rice starch with a certain amount of amylose as the donor substrate results in increases in the levels of crystallinity, solubility and paste clarity, gelatinization temperature (GT), enthalpy (Δ*H*), gel strength, and storage modulus (Lu et al., 2008; Guo et al., 2019). There is a reduction in the retrogradation of flour, a process in which disaggregated amylose and amylopectin chains in a gelatinized starch paste reassociate to form more ordered structures, when waxy rice flour is used, with an increase in AC (Sasaki et al., 2000). On the other hand, the fine structure of amylopectin is considered to affect the gelatinization, retrogradation, and rheology of waxy rice starch (Kalichevsky et al., 1990; Chung et al., 2008). During retrogradation of waxy rice starch, GT is positively correlated with the branch chain length of amylopectin (Jane et al., 1999; Singh et al., 2012), with the greater branch density of amylopectin being associated with increasing Δ*H* and solubility but with decreasing viscosity of waxy rice starch (Sorndech et al., 2015; Ren et al., 2017). These studies revealed the vital role played by amylopectin fine structure and AC in the physicochemical properties of waxy rice. However, there are no effective and rapid means for breeding waxy rice cultivars by targeting low AC or the fine structure of amylopectin, and this limitation has constrained the larger-scale production and consumption of waxy rice cultivars.

The *Wx* gene is located on rice chromosome 6 and encodes granule-bound starch synthase I (GBSSI), which mainly controls amylose synthesis in the seed endosperm and thus directly affects the quality of rice grains (Sano, 1984; Wang et al., 1995). The use of numerous allelic variants of *Wx* (e.g., *Wx^lv^*, *Wx^a^*, *Wx^in^*, *Wx*^b^, *Wx^op^*, *Wx^mp^*, and *wx*) in the rice breeding programs has led to regional differences in the AC of rice (Sano, 1984; Larkin and Park, 2003; Chen et al., 2008; Mikami et al., 2008; Dobo et al., 2010; Teng et al., 2011; Zhang et al., 2019) and allows rice breeders to control the expression of the *Wx* gene to obtain rice varieties with desired starch quality traits (Satoh and Omura, 1981; Liu et al., 2003; Liu et al., 2005; Kong et al., 2015). In recent years, the CRISPR/Cas9 gene-editing system has been used to knock out or fine-tune the expression of the *Wx* gene (Ma et al., 2015). The first exon of the *Wx* gene was edited to produce a null mutation *via* CRISPR/Cas9, to generate four *japonica* rice *wx* mutant lines with significantly reduced AC, but with an unaltered expression of agronomic traits (Zhang et al., 2018; Yunyan et al., 2019). Editing the TATA box of the *Wx^b^* promoter downregulated *Wx* expression and thus fine-tuned grain AC, which could improve the grain quality (Huang et al., 2020). However, the relationship between the physicochemical properties of rice starch in each *wx* mutant and its corresponding wild type (WT) remains unclear, and more information is needed before breeding for high-quality waxy starch by gene editing can be carried out effectively.

In the current study, we systematically analyzed the physicochemical properties of *wx* mutants generated in different genetic backgrounds by the CRISPR/Cas9 system. The results showed that the physicochemical properties of *wx* mutants are highly correlated with the properties of the WT parent, suggesting that parents with low initial AAC should be preferred for generating waxy rice mutants with desirable starch characteristics. Our work provides new insights into the breeding of waxy rice with high-quality starch and could contribute significantly to expanding the waxy rice germplasm resources.

## Results

### Mutant Isolation and Agronomic Trait Assessment

**FIGURE 1:**
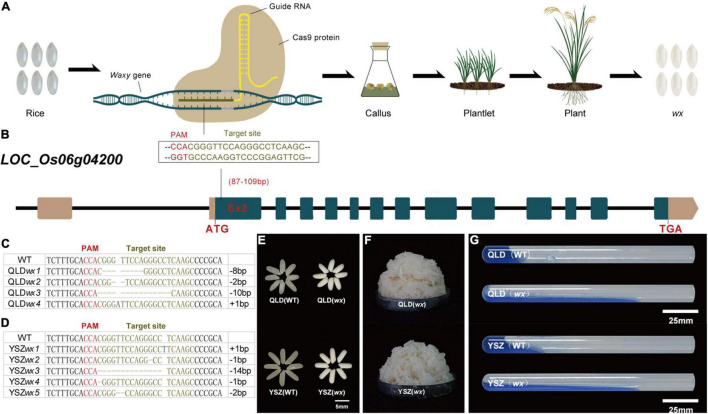
New waxy rice by CRISPR/Cas9-mediated mutagenesis of *Waxy* gene in two rice varieties. **(A)** Schematic experimental design. From left to right are wild-type rice, CRISPR/Cas9 system, callus, plantlet, plant, and waxy rice. **(B)** Schematic diagram of the targeted site in exon 2 of *Waxy* gene (*LOC_Os06g04200*). The numbers in brackets indicate the distance to the start codon (ATG). **(C–D)** Nucleotide variations at the targets (the protospacer adjacent motif in blank) of homozygous mutant lines from YSZ*wx1* and QLD*wx4.* “−”, base deletion; “+”, base insertion. The targeted sequence is highlighted in brown, and the protospacer adjacent motif (PAM) sequences are in red. **(E)** Grain phenotypes of *wx* mutants and their corresponding WTs. **(F)** The appearance of cooked waxy rice. **(G)** Gel consistency.

We first selected two cultivated rice varieties with different major *Wx* alleles, namely, QLD (*Wx^a^*) and YSZ (*Wx^b^*), which are both widely grown in the rice-producing region of Southwest China. We designed a CRISPR/Cas9 (Figure 1A) construct that accurately targeted the second exon (87–109 bp) of the *Wx* gene with the expectation of generating a null mutation (Figure 1B). In the genetic background of QLD (Figure 1C), we obtained four homozygous mutant alleles for the target site, with each of these mutations shifting the reading frame of the candidate gene in the 5’ coding region, indicating that they were most likely to be null alleles. Five homozygous mutant alleles of *Wx^b^* were also generated in the background of YSZ (Figure 1D). We observed that 80% of T_0_ transformants were mutants in QLD plants and 82.35% in YSZ plants, indicating that the CRISPR/Cas9 system exhibited a high mutagenesis efficiency (Supplementary Table 1). Meanwhile, our results showed that the *wx* mutations did not change the main agronomic traits of the T_1_ generation lines, including plant height, panicle number per plant, grain number per plant, and seed-setting frequency (Supplementary Figure 1).

### Assessment of the Grain Appearance and Cooking Quality of the *wx* Mutants

In addition, we identified two single-base homozygous mutants, YSZ*wx1* and QLD*wx4* (T_5_ generation), and performed quality analysis on them (Supplementary Table 2). The results showed no significant difference in the major grain quality traits of the two *wx* mutants, except that the color of endosperm changed from transparent to milky white (Figure 1E) and the kernel weight decreased (Supplementary Figure 2). The cooking and eating quality of YSZ*wx1* rice was soft but not sticky, while that of the QLD*wx4* rice was very sticky (Figure 1F), and the grains of both mutants exhibited a significantly higher gel consistency (GC) than that of flour of the corresponding WT parents (Figure 1G).

### Analyses of Apparent Amylose Content, Total Protein, Total Starch, and Grain Yield in *wx* Mutants

**FIGURE 2:**
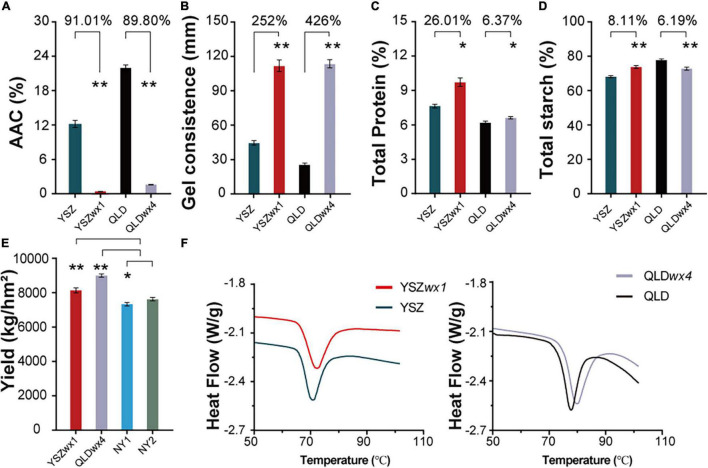
Apparent amylose content (AAC), gel consistency (GC), total protein, total starch, grain yield, and gelatinization properties of *wx* mutants and their corresponding WT lines. **(A)** AAC of *wx* mutants and their corresponding WTs. **(B)** GC of *wx* mutants and their corresponding WTs. **(C)** Total protein of *wx* mutants and their corresponding WTs. **(D)** Total starch of *wx* mutants and their corresponding WTs. **(E)** The grain yield of *wx* mutants of NY1 and NY2. NY1 is a *japonica* waxy rice (in blue), NY2 is an *indica* waxy rice (in green). **(F)** Gelatinization properties curve in *wx* mutants and their corresponding WTs. Error bars are mean ± SD (*n* = 3). Significant differences were determined by Student’s *t*-test (*P < 0.05, **P < 0.01).

We observed that the AAC values of YSZ and QLD were significantly decreased to 1.16 and 2.36%, respectively (Figure 2A) (reduced by 91.01 and 89.80%, respectively) in the corresponding mutants, leading to increased GC (Figure 2B). The total protein content of YSZ and QLD was increased by 26.01 and 6.37%, respectively, in the mutants (Figure 2C), but the total starch content showed no consistent effect in the mutants, increasing by 8.11% in the YSZ mutant and decreasing by 6.19% in the QLD mutant, compared with the corresponding WT (Figure 2D). Moreover, the rice yield values indicated that the grain yields of YSZ*wx1* and QLD*wx4* were 8,195 and 9,050 kg/hm^2^ (= kg/ha), respectively, which were significantly higher than the yields of the corresponding wild types (Figure 2E). These results suggest that editing the *Wx* gene can significantly improve both the quantity and the quality of the rice produced by the mutants.

### Analyses of Starch Thermal Properties of the Mutants

The results of analyses of the thermal properties of the starch from the *wx* mutants suggested that editing of the *Wx* gene did not significantly change the GT, although the two *wx* mutants showed a slightly higher GT range than the WT lines (Figure 2F). For example, the onset temperature (*To*), peak temperature *(Tp)*, conclusion temperature (*Tc*), and Δ*H* of YSZ and YSZ *wx1* were 66.28 and 66.65°C, 70.70 and 71.66°C, 76.27 and 78.74°C, and Δ*H* of 11.6 and 12.55 J/g, respectively (Supplementary Table 3). Similar results were also obtained for QLD and QLD*wx4* (Supplementary Table 3). The results suggest that *To*, *Tp*, *Tc*, and Δ*H* from the *wx* mutants were all higher, compared with the corresponding WT, which led to the mutant starch being gelatinized later than the WT starch. However, the mutant starch still belonged to the same GT type as the WT starch.

### Analyses of Starch Pasting Properties of the Mutants

**FIGURE 3:**
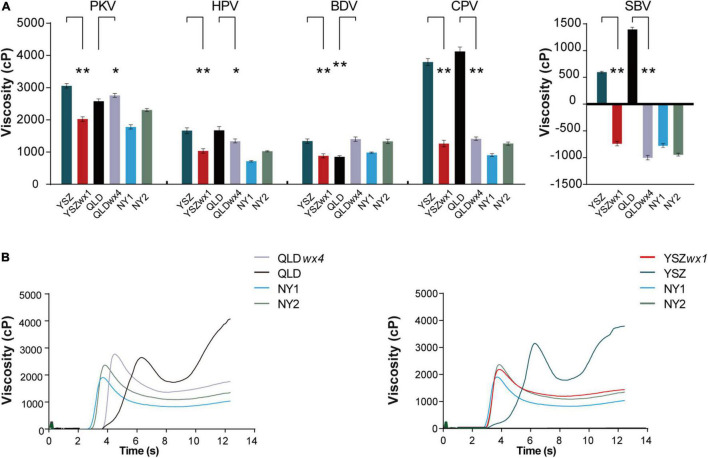
Rapid viscosity analyzer profiles of rice starch in *wx* mutants and their corresponding WTs. **(A)** Rapid viscosity analyzer (RVA) profiles of starch in *wx* mutants and their corresponding WTs. **(B)** Rapid viscosity analyzer profile curve of *wx* mutants and their corresponding WT flour samples. The date of peak viscosity (PKV), hot paste viscosity (HPV), cool paste viscosity (CPV), breakdown viscosity (BDV = PKV-HPV), and setback viscosity (SBV = CPV-HPV) were derived from the RVA profiles. NY1 is a *japonica* waxy rice variety (in blue), and NY2 is an *indica* waxy rice variety (in green). Error bars are mean ± SD (*n* = 3). Significant differences were determined by Student’s *t*-test (*P < 0.05, **P < 0.01).

The pasting properties of all *wx* mutants were significantly different from those of the corresponding WT lines (*P* < 0.05) (Figure 3A). For example, the mean peak viscosity (PKV), hot paste viscosity (HPV), cool paste viscosity (CPV), breakdown viscosity (BDV = PKV-HPV), setback viscosity (SBV = CPV-HPV), peak time, and pasting temperature (PT) of QLD and QLD*wx4* were 2,662 and 2,841 cp, 1,760 and 1,432 cp, 4,210 and 1,497 cp, 939 and 1,488 cp, 1,548 and -1,344 cp, 6.38 and 4.57 s, and 80.7 and 81.5°C, respectively (Supplementary Table 4). The results show that *wx* mutants have softer pasting properties than their corresponding WT lines (Figure 3B).

### Chain-Length Distribution of Amylopectin

**FIGURE 4:**
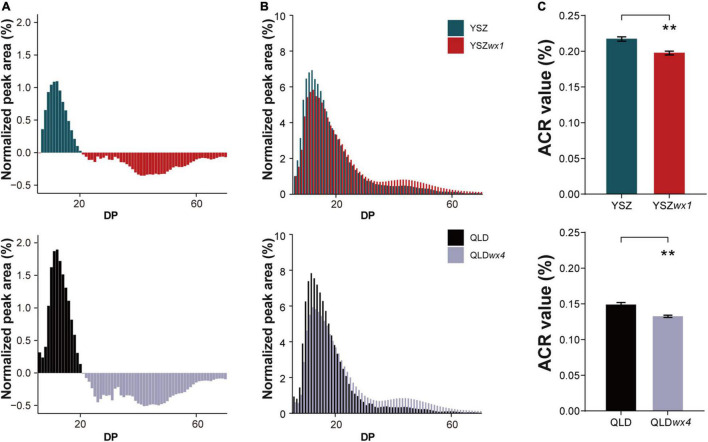
The fine structure of amylopectin in *wx* mutants and their corresponding WTs. **(A)** Comparison of percentage values of high-performance anion-exchange chromatography with pulsed amperometric detection (HPAEC-PAD) chromatograms of amylopectin chain length between *wx* mutants and their corresponding WTs. The plus values represent the WTs and the minus values represent the *wx* mutants. **(B)** The difference in the chain-length distribution of amylopectin between *wx* mutants and their corresponding WTs. **(C)** The ΣDP ≤ 10/ΣDP ≤ 24 values of *wx* mutants and their corresponding WTs. ACR: amylopectin chain ratio. Error bars are mean ± SD (*n* = 3). Significant differences were determined by Student’s *t*-test: **P < 0.01.

Subsequently, we compared the chain-length distribution of amylopectin between the two *wx* mutants and their corresponding WT parents. The chain-length distributions of amylopectin in *wx* mutants were markedly different from those of the corresponding WT lines (Figure 4A). *Wx* mutants exhibited a higher percentage of short-chain amylopectin (degree of polymerization, DP 7–20) and a lower percentage of medium- to long-chain amylopectin (DP > 20) than the WT lines (Figure 4B). Based on the amylopectin chain ratio (ACR) [ACR = (ΣDP ≤ 10)/(ΣDP ≤ 24)], the amylopectin structure of the starch from cultivated rice can be classified into one of three types: L-type (ACR ≤ 0.200), M-type (ACR 0.201–0.239), and S-type (ACR ≥ 0.240) (Nakamura et al., 2002). Interestingly, even though the ACR values of the *wx* mutants were significantly lower than those of the corresponding WT lines, they still belonged to the same amylopectin structural type. The amylopectin structure of QLD*wx4* and QLD belonged to the L-type, whereas YSZ*wx1* and YSZ belonged to the M-type (Figure 4C).

### Correlation Analysis of Starch Samples

**FIGURE 5:**
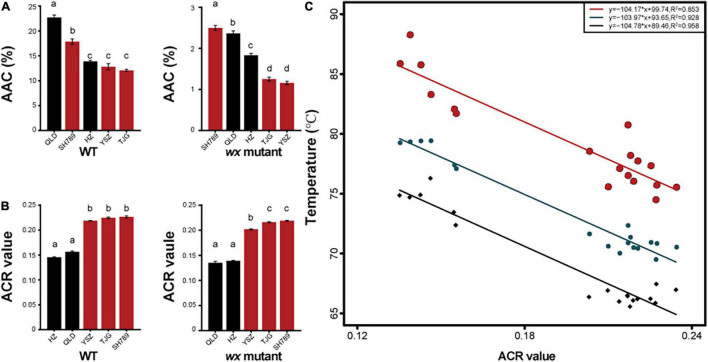
The apparent amylose content (AAC) and amylopectin chain ratio (ACR) value of five wx mutants and their corresponding WTs **(A)** AAC of five *wx* mutants and their corresponding WTs. **(B)** ACR value of five *wx* mutants and their corresponding WTs. AAC and ACR value of WTs (left-hand bar graph) and *wx* mutants (middle bar graph). **(C)** Relationships between ACR value and gelatinization temperature; each point represents the ACR value of a variety of rice or waxy rice. Black points represent the onset temperature (*To*), bluish-black points represent the peak temperature (*Tp*), and red points represent the conclusion temperature (*Tc*). Error bars are mean ± SD (*n* = 3). Values followed by different superscripts in the same column are significantly different (*P* < 0.05).

We used the same method for generating *wx* mutants with three other rice cultivars to find a potential link between gene-edited *wx* mutants and wild types (WTs) (Supplementary Figure 3 and Supplementary Table 5). These three rice cultivars all belonged to the *Wx^b^* genotype (Supplementary Table 6). The results showed that the cultivar (Figure 5A) with a low AAC would generate a *wx* mutant with a lower AAC (Figure 5B). Furthermore, the AAC of the *wx^a^* mutants was higher than that of the three *wx^b^* mutants, and the AAC values of QLD*wx4* and SH789*wx* were greater than 2% (Supplementary Tables 2, 6). The *Tc* and Δ*H* values of the *wx* mutants were greater than those of the corresponding WTs, although their GT types were unchanged (Table 1). Similarly, the ACR values were altered by mutation but stayed within the same amylopectin structure type as the WT (Figure 5B). Further data analysis showed a positive linear correlation between ACR and GT (Figure 5C). In addition, two new starch types from the mutants were proposed, based on the amylopectin structure and the GT, namely, the HGT-type (high gelatinization temperature type, ACR < 0.18) and LGT-type (low gelatinization temperature type, ACR > 0.18). We demonstrated that the GT type and ACR type were not affected by the editing of the *Wx* gene, and that, to achieve *wx* mutants with a lower AAC, the WT parent exhibiting a lower AAC should be selected.

**TABLE 1:** Differential scanning calorimetry (DSC) and enthalpy (Δ*H*) values of *wx* mutants and their corresponding WTs.

Cultivar	*To* (Δ)	*Tp* (°C)	*Tc* (°C)	Δ *H* (J/g)	*Tc – To* (°C)	GT type
YSZ	66.0 ± 0.1	70.3 ± 0.2	76.2 ± 0.2	11.9 ± 0.2	10.2 ± 0.2	*I*
YSZ*wx1*	66.4 ± 0.1**	71.8 ± 0.2**	78.6 ± 0.4**	13.3 ± 0.3**	12.2 ± 0.2**	
QLD	73.5 ± 0.5	77.3 ± 0.3	81.9 ± 0.3	12.8 ± 0.2	8.4 ± 0.3	*H*
QLD*wx4*	74.8 ± 0.5*	79.6 ± 0.4**	86.0 ± 0.3**	15.2 ± 0.4**	11.2 ± 0.8**	
SH789	65.9 ± 0.1	70.1 ± 0.7	74.6 ± 0.2	10.8 ± 0.2	8.8 ± 0.2	*I*
SH789*wx*	65.5 ± 0.2*	71.4 ± 0.2*	78.2 ± 0.2**	12.7 ± 0.3**	12.7 ± 0.1**	
TJG	66.3 ± 0.1	71.1 ± 0.2	77.6 ± 0.3	10.3 ± 0.2	11.3 ± 0.3	*H*
TJG*wx*	66.6 ± 0.1*	72.3 ± 0.2**	80.5 ± 0.2**	14.1 ± 0.2**	14.0 ± 0.3**	
HZ	76.3 ± 0.2	79.3 ± 0.3	83.6 ± 0.3	14.0 ± 0.2	7.2 ± 0.2	*H*
HZ*wx*	74.9 ± 0.2**	79.6 ± 0.4	88.5 ± 0.5**	15.4 ± 0.2**	13.6 ± 0.3	

To, onset gelatinization temperature; Tp, peak gelatinization temperature; Tc, final gelatinization temperature; ΔH, gelatinization enthalpy (J/g); I, intermediate gelatinization temperature type; H, high gelatinization temperature type. Data are presented as means l’ sd. *P < 0.05, **P < 0.01.

**FIGURE 6:**
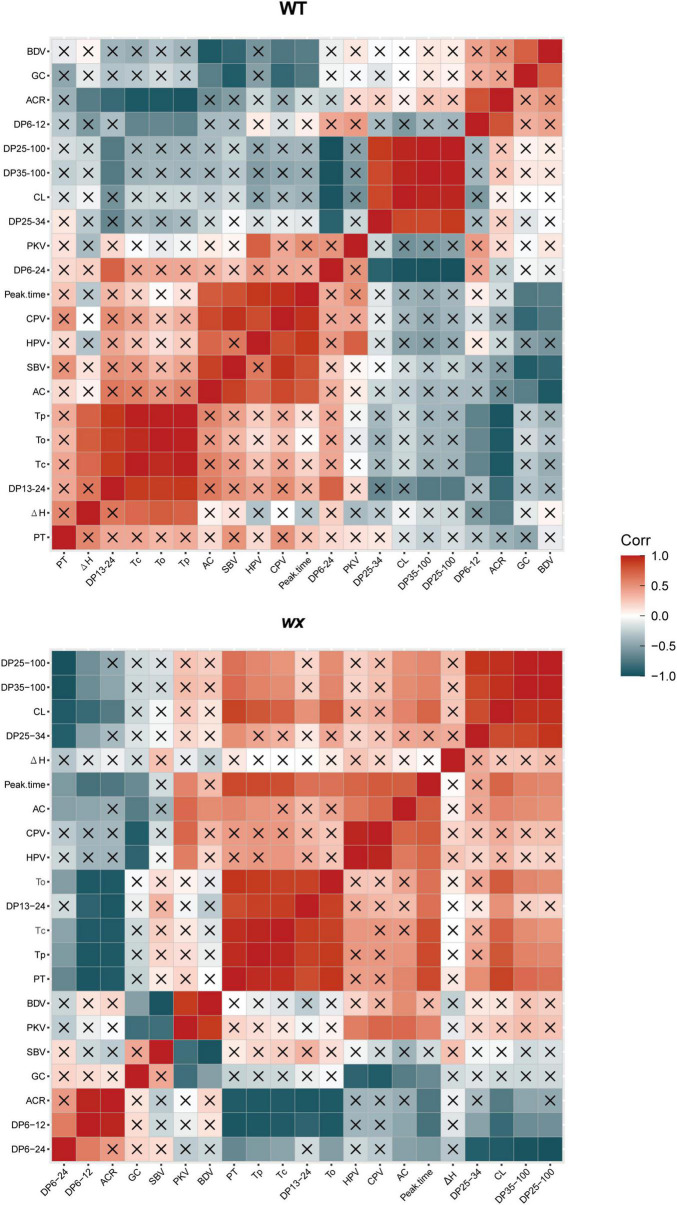
Pearson’s correlation analysis of the relationship between amylopectin structure of *wx* mutants and their corresponding WTs. The stronger the red color, the more significant the positive correlation; the stronger the bluish-black color, the more significant the negative correlation. “×” means no correlation. Δ*H*, gelatinization enthalpy; *To*, onset temperature; *Tp*, peak temperature; *Tc*, conclusion temperature; AC, amylose content; PKV, peak viscosity; HPV, hot paste viscosity; CPV, cool paste viscosity; BDV, breakdown viscosity; SBV, setback viscosity; PT, pasting temperature; ACR value, amylopectin chain ratio; CL, chain length; GC, gel consistency.

In this study, we also separately analyzed the relationship between the physicochemical properties of the starch obtained from the WT and the *wx* mutants. Pearson’s linear correlation analysis indicated that the correlations between rice grain quality, amylopectin structure, and starch physicochemical properties differed between WTs and the *wx* mutants (Figure 6). The WT data revealed that GT was negatively correlated with both DP 6–12 and ACR but was positively correlated with DP 13–24. The AAC was positively correlated with peak time, CPV, HPV, and SBV, but was negatively correlated with BDV and GC. On the other hand, the data from *wx* mutants indicated that GT was negatively correlated with DP 6–12, DP 6–24, and ACR, but was positively correlated with DP 13–24, peak time, CL, and DP 25–100. The AAC of *wx* mutants showed a negative correlation with GC and a positive correlation with peak time, HPV, and CPV. The results indicated that amylopectin structure, AAC, GT, and pasting properties in different rice cultivars reflected different physicochemical properties.

## Discussion

Over the past few decades, many waxy rice cultivars have been developed by conventional plant breeding techniques and mutagenesis. However, these methods have the disadvantages of unpredictable starch quality, as well as being time-consuming and laborious (Toda, 1980; Deng, 1992; Olsen and Purugganan, 2002). Recently, the development of genome-editing technology, particularly the discovery and application of the CRISPR/Cas9 gene-editing system, has led to its widespread use in studying plant genome functions (Ran et al., 2013; Belhaj et al., 2015; Bortesi and Fischer, 2015). Several studies have reported that the *Wx* gene can be successfully edited with high efficiency in rice through the use of the CRISPR/Cas9 system, which shows a high average mutation rate of up to 80%, without affecting the major agronomic traits in the transgenic lines (Zhang et al., 2018; Yunyan et al., 2019; Huang et al., 2020). In agreement with previous studies, the *wx* mutation rate in the present study showed a high value of 80% and above, and *wx* mutants exhibited major agronomic traits similar to those of their corresponding WT lines. However, in this study, we identified the *Wx* allele and the starch quality of the WT lines before editing and compared the major starch quality traits of the *wx* mutants from parents with different *Wx* alleles, an approach that is different from that undertaken in previous studies.

Our results showed that the AAC values of three *wx^b^* mutants were lower than that of *wx^a^* mutants (Supplementary Tables 2, 6), and WT parents with a lower level of AAC will generate *wx* mutants with lower AAC values. The AAC of *wx^a^* mutants was higher than the 2% upper threshold, a characteristic that is widely believed to be unacceptable for the AAC of elite waxy rice grains (Juliano, 1992). Therefore, our results indicate that it is necessary to select parental rice varieties with a lower level of AAC to obtain high-quality waxy rice grains in the breeding program (conventional or mutagenesis breeding). A recent study reported that silencing the expression of the *Wx* gene regulates genes related to amylose synthesis, with upregulation of granule-bound starch synthase II (GBSSII) compensating for some loss of amylose in the rice endosperm (Pérez et al., 2019). This may explain why the same *Wx* parental genotype results in different AAC levels after gene editing (Figure 5A), and further studies are warranted to study the contribution of different GBSSII types to the amylose content in endosperm in order to develop new waxy rice varieties with more precise and desirable AAC levels.

Amylopectin is also proposed to have a significant influence on the physicochemical properties of waxy rice flour (Man et al., 2013; Huang and Lai, 2014). Previous studies had shown that some starch-synthesizing enzymes influence the structure of amylopectin, such as soluble starch synthases (SSs), branching enzymes (BEs), debranching enzymes (DBEs), and isoamylase 1 (ISAI) (Umemoto et al., 1995; Man et al., 2013; Shufen et al., 2019; Zhu et al., 2020). These observations may rationally explain the differences in chain-length distributions of amylopectin in different *wx* mutants obtained from the same *Wx* allele (Figures 4, 5 and Supplementary Figure 4). The expression of the major starch synthesis-related genes is upregulated following the silencing of the expression of the *Wx* gene (Pérez et al., 2019), leading to the differences in chain-length distributions of amylopectin observed in *wx* mutants and their corresponding WTs (Figure 4 and Supplementary Figure 4). Although the chain-length distributions of amylopectin were changed, the range of ACR values was not significantly different (Figure 5). Based on the ACR values, three types of amylopectin could be distinguished, namely L-type (ACR < 0.2), M-type (0.2–0.24), and S-type (ACR > 0.24). The GT of rice can also be divided into three types: low-type (<70°C), intermediate-type (70–74°C), or high-type (>74°C), and is negatively correlated with ACR (Kongseree and Juliano, 1972; Nakamura et al., 2002; Vandeputte et al., 2003). In the current study, we found that *wx* mutants exhibited ACR values and amylopectin types similar to those of their corresponding WTs, and that the ACR value was linearly correlated with GT, which means that the breeder can obtain a waxy rice genotype with a GT and an amylopectin structural type similar to those of the corresponding WT. We also distinguished two amylopectin structure types based on ACR values: HGT-type (ACR < 0.18) and LGT-type (ACR > 0.18). The GT of the HGT-type is more than 77°C, in contrast to that of the LGT-type, which is less than 72°C (Table 1). Collectively, it can be easier to discriminate between amylopectin structure types selected on the basis of GT, which could benefit breeding for rice with specific waxy starch characteristics.

Previous studies had tended to indicate that, in all rice cultivars, the GT was negatively correlated with short amylopectin chains (DP 6–12) and ACR values, but positively correlated with the medium (12 ≤ DP ≤ 24) and long amylopectin chains (DP > 37) (Wang and Wang, 2002; Vandeputte et al., 2003; Wong et al., 2003). However, our study indicated that GT was only significantly negatively correlated with ACR and DP 6–12 when the genotypes were WTs, but was significantly positively correlated with DP 25–100 when all were *wx* mutants or waxy rice genotypes (Figure 6). Therefore, carrying out a statistical analysis of starch quality data combined from both non-waxy and waxy genotypes may not be the best way to identify a relationship between the physicochemical properties. On the basis of the above-mentioned results, we suggest that analyzing the relationship between the physicochemical properties of the same variety (waxy or non-waxy) can provide greater accuracy, and that selection on the basis of ACR value can more effectively identify genotypes with specific GT values.

## Conclusion

In the current study, five new waxy rice mutants were generated, using the CRISPR/Cas9 system to edit the *Wx* gene. Detailed analysis of the two *wx* mutants from QLD and YSZ showed a significant decrease in AAC and viscosity compared with the corresponding WT, and a significant increase in GC and total protein content, with the grain yields of both mutants being higher than those achieved by the two parental elite cultivated rice WTs. Based on the ACR value, two types of amylopectin structure are proposed: HGT-type (ACR < 0.18; GT > 77°C) and LGT-type (ACR > 0.18; GT < 72°C). Our study also found that WTs with a low level of AAC generated *wx* mutants with lower AAC, with the *wx* mutants also having values of ACR, GT, and amylopectin structure types similar to those of their corresponding WTs, suggesting that parents with targets of specific GT values and low initial AAC should be selected for in breeding programs. In conclusion, we propose a novel strategy for generating waxy rice genotypes directly, and provide new insights into expanding waxy rice germplasm resources for use in breeding programs.

## Materials and Methods

### Plant Materials and Growth Conditions

We selected five elite rice varieties with different genetic backgrounds, including four *indica* varieties (YSZ, QLD, SH789, and HZ) and one *japonica* variety (TJG), which were mainly planted in eastern Asia, central China, and western China. The seed was supplied by the Rice Research Institute of Sichuan Agricultural University. The *japonica* waxy line NY1 and the *indica* waxy line NY2 were used as controls. Unless indicated, all rice lines were grown in paddy fields in Chengdu, China, during the normal rice-growing seasons.

### CRISPR/Cas9 Gene-Editing and Gene Cloning

The CRISPR/Cas9-targeted genome editing tool was constructed and operated as previously described (Feng et al., 2013). The primer sequences used to construct the vector are shown in Supplementary Table 7.

The polymerase chain reaction (PCR) amplification conditions were as follows: initial denaturation at 94°C for 2 min, followed by 35 cycles of denaturation at 94°C for 30 s, annealing at 56°C for 30 s, extension at 72°C for 30 s, followed by a final extension at 72°C for 5 min, with the PCR mix being based on the Foregene PCR Fast Mix. The PCR products were sequenced by the Sanger method. The primers used for *Wx* sequences, the *Wx* allele genotype, target sequences, and reporter gene detection are listed in Supplementary Table 7.

### Tasting Properties of Waxy Rice

A sample (30 g) of milled waxy rice grains was cooked in an electric rice cooker to determine the tasting properties (model MDFBZ02ACM, Xiaomi, China). The tasting quality of milled waxy rice was determined with an RCTA-11A Taste Analyzer (Satake, Japan) (Zhang et al., 2016).

### Measurements of Waxy Rice Starch

Grains of a single *wx* mutant with stable inheritance in the T_5_ generation were dried at 37°C for 2 weeks. The dried grains were shelled, polished, and milled by a pearling rice mill, and finally screened through a 74-μ mesh. Starch was prepared by the method of Precha-Atsawanan et al. (2018). The waxy flour was steeped in 0.35% sodium hydroxide solution and incubated at 4°C for 48 h. The precipitants in the suspension were collected by centrifugation at 3,000 g for 15 min. The starch layer was re-mixed with water to which was added 1% alkaline protease (Sigma), and the pH was adjusted to 9 with sodium hydroxide and incubated at 37°C for 8 h. The washing step was repeated at least three times or until the pH value reached 7. The final precipitant was collected by centrifuging at 3,000 g for 30 min, and the starch was dried in a warm air oven at 40°C for 48 h. The starch was ground with a mortar and pestle, using a 0.08-mm sieve to collect the starch powder.

### Quantification of Starch and Amylose

The rice flour was equilibrated in a constant temperature and humidity cabinet for 7 days. The starch content was determined according to the method of Smith and Zeeman (2006). D-glucose was stained with the GOPOD reagent (glucose oxidase plus peroxide and 4-aminoantipyrin) and determined by absorbance at 510 nm. Amylose content was measured as described in GB/T 15683-2008/ISO 6647-1: 2007. The amylase-iodine blue color was determined at 720 nm. Standard curves were plotted with standardized samples of rice amylose and amylopectin (China National Rice Research Institute, Zhejiang, China).

### Quantification of Gel Consistency and Total Protein

The gel consistency (GC) of grains was evaluated as described previously (Cagampang et al., 1973). A sample (100 ± 1 mg) of the starch powder was placed into a 13 × 100 mm tube and wetted with 0.2 ml of 95% ethanol containing 0.025% thymol blue, and then 2.0 ml of 0.2 M KOH was added and allowed to boil for 3 min. GC was measured in ml as the length (mm) of the gel spreading in the tubes when laid flat on the graph for 1 h. The total protein content of the grain was assayed by the Kjeldahl method according to the Comin amylose assay procedure (Suzhou Comin Biotechnology Co., Ltd).^1^

### Waxy Rice Powder Pasting Properties

To the white rice flour (3 g at 14% moisture content) obtained from the T_5_ transformant generation was added 25 ml of ddH_2_O_2_. The setting procedure included the following steps: incubation for 1 min at 50°C, increasing the temperature at a rate of 12°C/min to 95°C (over 3.75 min), maintaining the temperature at 95⋅C (for 2.5 min), then decreasing it to 50°C (over a period of 3.75 min) at 12°C/min, and maintaining the temperature at 50°C (for 2 min). The initial speed was set to 960 r/min for 10 s and then adjusted to 160 r/min. The pasting parameters of waxy rice flour, such as the peak viscosity (PKV), hot paste viscosity (HPV), cool paste viscosity (CPV), breakdown viscosity (BDV = PKV − HPV), setback viscosity (SBV = CPV − HPV), and pasting temperature (PT), were evaluated using a rapid viscosity analyzer (RVA, Newport Scientific, Australia) according to the methods of Chung et al. (2011).

### Thermal Properties of Waxy Rice Starch

The thermal properties of waxy rice starch were measured using differential scanning calorimetry (DSC Q2000; TA Instruments Ltd., Crawley, United Kingdom) (Yang et al., 2018). The onset temperature (*To*), peak temperature (*Tp*), conclusion temperature (*Tc*), and gelatinization enthalpy (Δ*H*) were recorded using a Universal Analysis 2000 (TA Instruments Ltd., Crawley, United Kingdom). A sample (3 mg) of starch was mixed with 6 μl of distilled water, and the paste was added to an aluminum pan and incubated at 4°C for 24 h. The specimens were then heated from 30 to 100°C at a rate of increase of 10°C/min.

### Amylopectin Chain-Length Distribution

The chain-length distribution of amylopectin was analyzed according to the method of Kowittaya and Lumdubwong (2014). Waxy rice starch was suspended in 95% (v/v) ethanol and boiled for 60 min. Hydrolyzed polyglucan was treated with *Pseudomonas amyloderamosa* isoamylase (1,000 U/μl, Sigma). The chain-length distribution of waxy rice starch was analyzed by high-performance anion-exchange chromatography with pulsed amperometric detection (HPAEC-PAD). There were three mobile phases: A (aqueous solution), B (100 mM NaOH and 1 M NaAC), and C (100 mM NaOH). The flow rate was controlled at 0.4 ml/min, and the column temperature was controlled at 30°C.

### Data Analysis

All experiments were independently repeated three times. The results were expressed as the mean ± standard deviation. The correlation was assessed using Pearson’s correlation coefficient. Data analysis was performed with SPSS 25.0, and the significance was defined as *P* < 0.05 (* or lowercase letters) and *P* < 0.01 (** or uppercase letters). SnapGene 5.2 was used to visualize PCR and cloning.

## Data Availability Statement

The original contributions presented in the study are included in the article/Supplementary Material, further inquiries can be directed to the corresponding author.

## Author Contributions

YF, TL, and JZ designed the strategy. YH, XY, XL, YL, YPL, BZ, RL, and ZZ completed part of the experiments. YF and JZ organized the figures and article modification. YF, TL, and JZ analyzed the data and wrote the manuscript. All authors commented on the manuscript.

## Conflict of Interest

The authors declare that the research was conducted in the absence of any commercial or financial relationships that could be construed as a potential conflict of interest.

## Publisher’s Note

All claims expressed in this article are solely those of the authors and do not necessarily represent those of their affiliated organizations, or those of the publisher, the editors and the reviewers. Any product that may be evaluated in this article, or claim that may be made by its manufacturer, is not guaranteed or endorsed by the publisher.

## Footnotes

1
www.cominbio.com
