# Supplementary material for: Assessment of the Characteristics of Waxy Rice Mutants Generated by CRISPR/Cas9
Source: Front Plant Sci. 2022 Jun 10;13:881964. doi: 10.3389/fpls.2022.881964 (PMC9226628; doi:10.3389/fpls.2022.881964)
Supplement: Supplementary file 1 [file Data_Sheet_1.docx]

**Table S1**. Percentage of T0 plants with mutations in the target locus

| **Cultivar** | **Target gene** | **No. of T0 plants examined** | **No. of plants with**  **mutations** | **Mutation rate (%)** |
| --- | --- | --- | --- | --- |
| YSZ | *Wx^a^* | 15 | 12 | 80 |
| QLD | *wx^b^* | 17 | 14 | 82.35 |

**Table S2*.*** *Waxy* alleles and mature seed quality of *wx* mutants and their corresponding WT lines

| **Cultivar** | **Genotype** | **TP (%)** | **AAC (%)** | **TS (%)** | **GC (mm)** |
| --- | --- | --- | --- | --- | --- |
| YSZ | *Wx^b^* | 8.01±0.17 | 12.81±0.61 | 70.51±0.62 | 48.00±2 |
| YSZ*wx1* | *wx^b^* | 10.09±0.37** | 1.16±0.04** | 76.23±0.80** | 115.33±5.03** |
| QLD | *Wx^a^* | 6.56±0.13 | 22.70±0.5 | 80.173±0.77 | 29.00±1.73 |
| QLD*wx4* | *wx^a^* | 6.98±0.11* | 2.36±0.07** | 75.21±0.92** | 117.00±3.61** |

TP, AAC, TS and GC = total protein, apparent amylose content, total starch, and gel consistency.

mm, millimeters. Data are presented as means ± sd. *P <0.05, **P < 0.01.

**Table S3**. Differential scanning calorimetry (DSC) of *wx* mutants and their corresponding WT lines

| **Cultivar** | **To (℃)** | **Tp (℃)** | **Tc (℃)** | **∆H (J/g)** |
| --- | --- | --- | --- | --- |
| YSZ | 66.28±0.19 | 70.70±0.34 | 76.27±0.22 | 11.83±0.26 |
| YSZ*wx1* | 66.65±0.33 | 71.66±0.45* | 78.74±0.33** | 13.54±0.26** |
| QLD | 73.53±0.56 | 77.73±0.37 | 82.06±0.50 | 12.70±0.17 |
| QLD*wx4* | 74.41±0.40 | 78.94±0.35* | 86.16±0.33** | 15.51±0.31** |

Onset temperature (To), peak temperature (Tp), conclusion temperature (Tc), and gelatinization enthalpy (ΔH). Data are presented as means ± sd. *P< 0.05, **P < 0.01.

| **Cultivar** | **PKV (cp)** | **HPV (cp)** | **CPV (cp)** | **BDV (cp)** | **SBV (cp)** | **Peak time(min)** | **PT (℃)** | |
| --- | --- | --- | --- | --- | --- | --- | --- | --- |
| YSZ | 3141±68 | 1754±91 | 3881±107 | 1428±68 | 740±90 | 6.34±0.05 | | 89.1±0.2 |
| YSZ*wx1* | 2113±71** | 1122±68** | 1347±102** | 972±63** | -766±160** | 3.88±0.01** | 72.7±0.25** | |
| QLD | 2662±81 | 1760±128 | 4210±135 | 939±35 | 1548±184 | 6.38±0.05 | 80.7±0.25 | |
| QLD*wx4* | 2841±63* | 1423±63** | 1497±54** | 1488±69** | -1344±51** | 4.57±0.04** | 81.2±0.26 | |
| NY1 | 1866±66 | 801±22 | 989±45 | 1070±18 | -877±70 | 3.74±0.03 | 71.5±0.22 | |
| NY2 | 2390±101 | 1113±33 | 1352±51 | 1418±75 | -1038.33±81 | 3.89±0.03 | 72.7±0.25 | |

**Table S4.** RVA profile characteristics of *wx* mutants and their corresponding WT lines

PKV, HPV, CPV, BDV, SBV and PT = peak viscosity, hot paste viscosity, cool paste viscosity, breakdown viscosity (BDV=PKV-HPV) and pasting temperature. cp, centipoise. Data are presented as means ± sd. *P <0.05, **P < 0.01.

**Table S5**. Agronomic and yield traits in additional three wx mutants and their corresponding WT plants

| **Cultivar** | **Plant height (cm)** | **panicle number per plant** | **Grain number per plant** | **seed-setting rate (%)** | **Grain width (mm)** | **Grain**  **length**  **(mm)** | **1000 grain weight**  **(g)** |
| --- | --- | --- | --- | --- | --- | --- | --- |
| SH789 | 125.1±4.1 | 10±1 | 165±12 | 95.6±0.1 | 2.734±0.02 | 10.18±0.12 | 29.15±0.3 |
| SH789*wx* | 124±3.2 | 11±1 | 162±8 | 96.7±0.3 | 2.753±0.10 | 9.92±0.23 | 25.15±0.4** |
| HZ | 105±3.0 | 11±1 | 286±15 | 97.3±0.6 | 2.886±0.01 | 9.105±0.08 | 21.30±0.5 |
| HZ*wx* | 102±3.5 | 11±1 | 296±6 | 97.5±1.0 | 2.856±0.03 | 9.143±0.13 | 17.50±0.2** |
| TJG | 95.2±2.4 | 6±2 | 125±7 | 93.3±0.7 | 3.21±0.12 | 7.32±0.10 | 22.65±0.3 |
| TJG*wx* | 94.5±3.2 | 7±1 | 120±3 | 94.5±0.2 | 3.17±0.08 | 7.38±0.13 | 18.05±0.2** |

cm, centimeter; mm millimeters; g gram Data are presented as means ± sd. **P < 0.01.

**Table S6*.*** Apparent amylose content (AAC), gel consistency (GC) and amylopectin chain ratio (ACR) values of *wx* mutants and their corresponding WT lines

| **Cultivar** | **Genotype** | **AAC (%)** | **GC (mm)** | **ACR** |
| --- | --- | --- | --- | --- |
| SH789 | *Wx^b^* | 17.64±0.37 | 32.3±1.5 | 0.23 |
| SH789*wx* | *wx^b^* | 2.53±0.03** | 89.7±2.1** | 0.22 |
| HZ | *Wx^b^* | 13.69±0.01 | 92.3±2.5 | 0.15 |
| HZ*wx* | *wx^b^* | 1.87±0.01** | 110.0±2.0** | 0.14 |
| TJG | *Wx^b^* | 12.38±0.06 | 81.0±0.2 | 0.22 |
| TJG*wx* | *wx^b^* | 1.19±0.04** | 130.3±2.5** | 0.22 |

AAC, GC and ACR = apparent amylose content, gel consistency and ΣDP≤10/ΣDP≤24 (the amylopectin chain ratio).

mm, millimeters. Data are presented as means ± sd. *P <0.05, **P < 0.01.

**Table S7.** Primers used in this study.

| **Primers** | **Forward primer** | **Reverse primer** |
| --- | --- | --- |
| Target1-412 | TGCAGAGATCTTCCACAGCA | TTGAAGACGACGACGGTCAG |
| Cas9-415 | CACCATCTACCACCTGAGAA | CGAAGTTGCTCTTGAAGTTG |
| Wx-1 | CCGTTGCGTCGTCATAGACAA | CCAGCCCAACACCTTACAGAAA |
| Wx-2  Wx-3  Wx-4 | GACAAGTACATCACCGCCAAGT  CGTTCGCTCACTGCTTCA  CATCCACAACATCTCCTACCA | ACAAGGTGGTGGACTAGACGAT  GTCACTCATTCTTGCCTTGTC  TTACCTCCACAGCCATAAGC |
