# Supplementary material for: Assessment of the Characteristics of Waxy Rice Mutants Generated by CRISPR/Cas9
Source: Front Plant Sci. 2022 Jun 10;13:881964. doi: 10.3389/fpls.2022.881964 (PMC9226628; doi:10.3389/fpls.2022.881964)
Supplement: Supplementary file 2 [file Data_Sheet_2.docx]

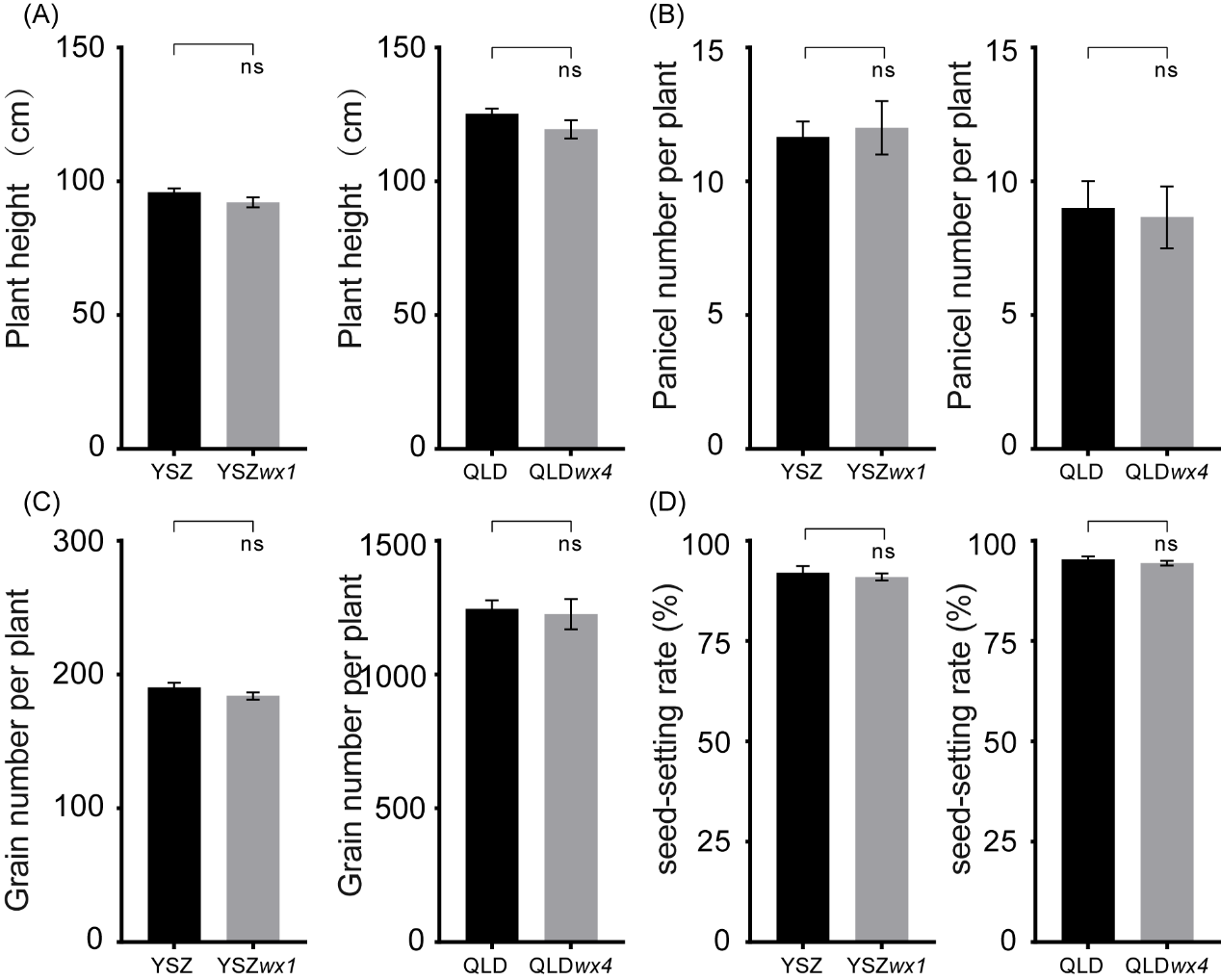


**Figure S1.** Plant height, panicle number per plant, grain number per panicle and seed-sitting rate in *wx* mutants and their corresponding WT plants

(a) Plant height of *wx* mutants mutant and corresponding their WTs. (b) panicle number per plant of *wx* mutants and corresponding their WTs. (c) grain number per panicle of *wx* mutants and corresponding their WTs. (d) seed-sitting rate of *wx* mutants and corresponding their WTs.

Data are presented as means ±sd. n=50 in a-d; two-tailed, two-sample Student t-test. NS, no significant


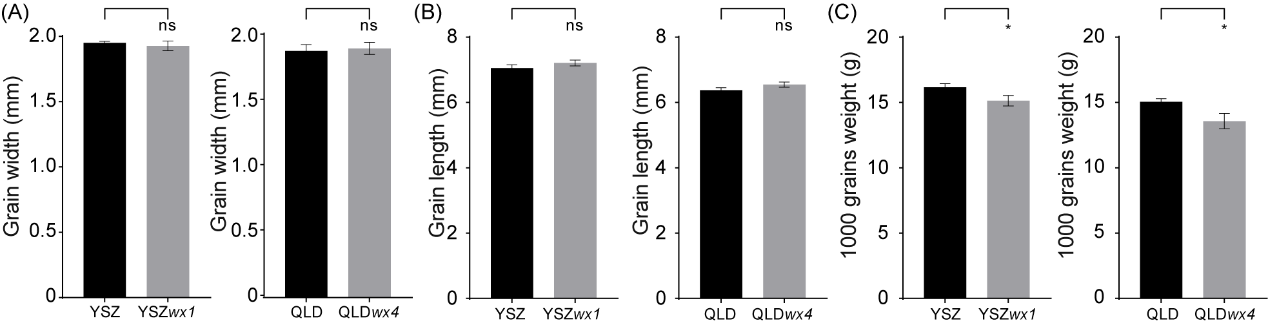


**Figure S2.** Grain width, grain length and 1000 grains weight in *wx* mutants and their corresponding WT plants

(a) Grain width of *wx* mutants and corresponding their WTs. (b) grain length of *wx* mutants and corresponding their WTs. (c) 1000 grains weight of *wx* mutants and corresponding their WTs.

Data are presented as means ±sd. n=50 in a-d; two-tailed, two-sample Student t-test. NS, no significant, *P < 0.05


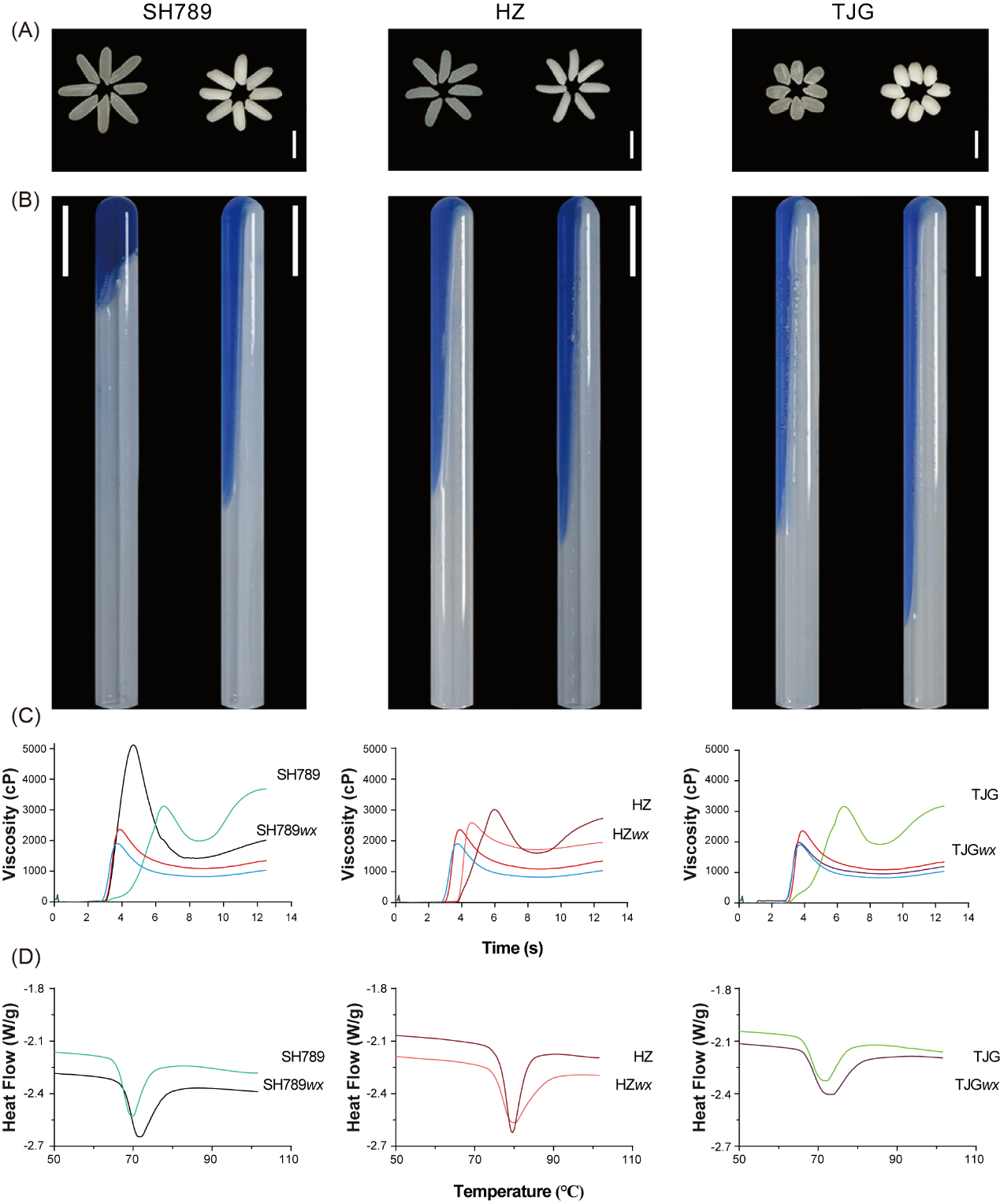


**Figure S3.** Grain phenotypes, gel consistency, rapid viscosity analyzer profiles and gelatinization properties in *wx* mutants and their corresponding WT lines

(a) Grain phenotypes of *wx* mutants and corresponding their WTs. (b) Gel consistency of *wx* mutants and corresponding their WTs. (c) Rapid viscosity analyzer profiles curve of *wx* mutants and corresponding their WTs. (d) Gelatinization properties curve of *wx* mutants and corresponding their WTs.

**
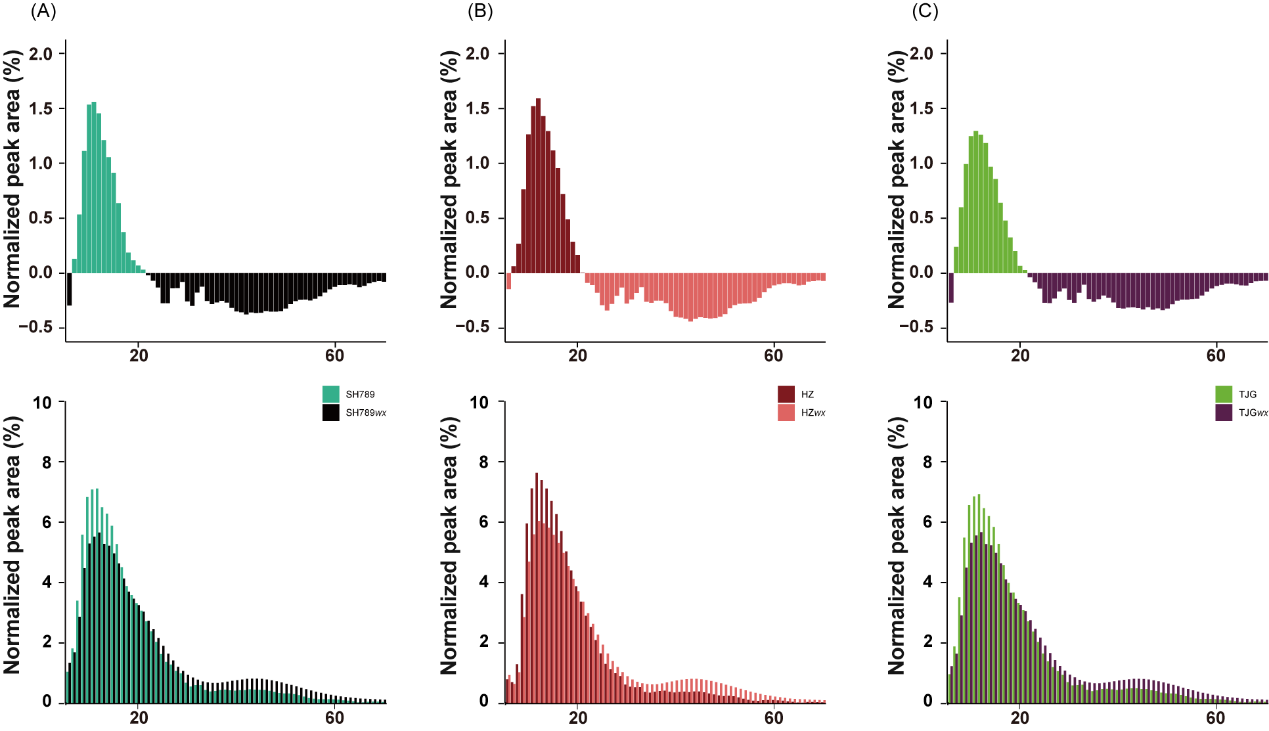
Figure S4.** The fine structure of amylopectin in wx mutants and their corresponding WTs

(a-c) The top row of the comparison of percentage values of high-performance anion exchange chromatography with pulsed amperometric detection (HPAEC-PAD) chromatograms of amylopectin chain-length between *wx* mutants and their corresponding WTs. The plus values represent the WTs and the minus values represent the *wx* mutants. The bottom row of difference in the chain-length distribution of amylopectin between *wx* mutants and their corresponding WTs.
